# Knockdown siRNA targeting GPR55 reveals significant differences between the anti-inflammatory actions of KLS-13019 and cannabidiol

**DOI:** 10.21203/rs.3.rs-3982851/v1

**Published:** 2024-02-28

**Authors:** Douglas E. Brenneman, William A. Kinney, Mark E. McDonnell, Michael J. Ippolito, Sara Jane Ward

**Affiliations:** Kannalife Sciences, Inc* Pennsylvania Biotechnology Center; Kannalife Sciences, Inc* Pennsylvania Biotechnology Center; Kannalife Sciences, Inc* Pennsylvania Biotechnology Center; Temple University; Temple University

**Keywords:** dorsal root ganglion, neuroinflammation, GPR55, NLRP3, IL-1beta, mitochondria

## Abstract

KLS-13019 was reported previously to reverse paclitaxel-induced mechanical allodynia in a mouse model of chemotherapy-induced peripheral neuropathy (CIPN). Recent studies demonstrated that paclitaxel-induced increases in inflammatory markers (GPR55, NLRP3 and IL-1b) of dorsal root ganglion (DRG) cultures were shown to be reversed by KLS-13019 treatment. The mechanism of action for KLS-13019-mediated reversal of paclitaxel-induced neuroinflammation now has been explored using GPR55 siRNA. Pretreatment of DRG cultures with GPR55 siRNA produced a 21% decrease of immunoreactive (IR) area for GPR55 in cell bodies and a 59% decrease in neuritic IR area, as determined by high content imaging. Using a 24-hour reversal treatment paradigm, paclitaxel-induced increases in the inflammatory markers were reversed back to control levels after KLS-3019 treatment. Decreases in these inflammatory markers produced by KLS-13019 were significantly attenuated by GPR55 siRNA co-treatment, with mean IR area responses being attenuated by 56% in neurites and 53% in cell bodies. These data indicate that the percentage decreases in siRNA-mediated attenuation of KLS-13019-related efficacy on the inflammatory markers were similar to the percentage knockdown observed for neuritic GPR55 IR area. Similar studies conducted with cannabidiol (CBD), the parent compound of KLS-13019, produced low efficacy (25%) reversal of all inflammatory markers that were poorly attenuated (29%) by GPR55 siRNA. CBD was shown previously to be ineffective in reversing paclitaxel-induced mechanical allodynia. The present studies indicated significant differences between the anti-inflammatory properties of KLS-13019 and CBD which may play a role in their observed differences in the reversibility of mechanical allodynia in a mouse model of CIPN.

## Introduction

Neuropathic pain is a pathological condition characterized by spontaneous pain, hyperalgesia and allodynia. Clinical data indicate that approximately 50% of treated individuals were unresponsive to current pharmacotherapies, and in those that receive some benefit, pain relief was typically incomplete (Bonezzi and Demartini, 1999). Of particular therapeutic interest to us was neuropathic pain associated with chemotherapy-induced peripheral neuropathy (CIPN). In this adverse effect produced by many chemotherapeutic regimens, 30–40% of patients experience a progressive, enduring, and sometimes irreversible condition featuring pain, numbness, tingling and sensitivity to cold in the hands and feet (Gutierrez-Gutierrez et al 2010). In many cases, this neuropathic pain associated with CIPN can result in a dose-limiting side effect which, in severe cases, can progress to an irreversible condition (Argyriou et al., 2014). Associated effects on peripheral nerves can lead to oxidative stress and inflammation, sensitization and spontaneous activity of peripheral nerve fibers, and hyperexcitability in the dorsal column of the spinal cord leading to ascending pain pathway sensitization (Peters et al 2007). In addition to neuronal responses, Schwann cells and microglia respond to chemotherapy by stimulating the release of substances that enhance excitability which may contribute to pain hypersensitivity (Koyanagi et al.,2021; Shan et al., 2024).

With neuropathic pain being unresponsive to current therapies, the use of medical cannabis and CBD has achieved an anecdotal level of acceptance by caregivers. To date, the treatment for neuropathic pain by CBD has lacked a demonstration of efficacy in a large, randomized clinical trial. However, a small clinical study of oral CBD has been reported recently to attenuate early symptoms of CIPN in a trial that utilized CBD *pretreatment* prior to the initiation of chemotherapy (Nielsen et al., 2022). There have been no reports of a reversal of CIPN in any human trial with any drug, including cannabinoids. While verifiable human data has yet to be achieved, significant efficacy has been reported in animal models of CIPN focused on mechanical and cold allodynia in rodents treated with paclitaxel (Ward et al., 2014; King et al., 2017). These studies, which have focused on the ability of CBD to *prevent* the onset of allodynia associated with paclitaxel treatment, provided supportive evidence in a reproducible model that has driven our interest in this therapeutic area.

Despite the promising efficacy of CBD in *preventing* CIPN, the pharmacological limitations of this compound have been concerning: low potency, reduced efficacy, liver safety, and oral bioavailability (Brenneman et al., 2018; Foss et al, 2021). With the recognition that optimization of CBD was warranted, the development of CBD analogues was undertaken with the eventual emergence of KLS-13019 (Kinney et al, 2016), a novel compound which has been shown to be effective in both the *prevention* and reversal of allodynia in a mouse model of CIPN (Foss et al, 2021). Because of the previously demonstrated overlap of preventative efficacy in a mouse model of CIPN, these two cannabinoids (CBD and KLS-13019) were chosen to compare for their effects on *reversing* inflammation.

While various animal models of CIPN have shown that peripheral and central glial cells exhibit increased pro-inflammatory responses (Lees et al, 2017), our studies have shown that paclitaxel treatment also increases pro-inflammatory responses in DRG neurons, indicating an end point targeting for inflammatory activation of this cell type as well (Brenneman et al., 2022). Because of the suggested importance of inflammation in CIPN (Makker et al., 2017; Fumagalli et al., 2021), the focus of our studies pivoted to selecting relevant molecular targets for inflammation in neurons. As with the reported neuroprotective effects mediated by regulation of mNCX-1 (Brenneman et al., 2019), the strategy for selecting target candidates relevant to inflammation was based on CBD and endocannabinoid pharmacology (Bih et al., 2015; Guerrero-Alba et al, 2019). GPR55 has been described as an endocannabinoid GPCR associated with pain and inflammation (Staton et al., 2008). More recently, a study has reported positive effects of GPR55 receptor antagonism in a rodent model of formalin-induced inflammatory pain (Okine et al., 2020). Thus, with the focus of our mechanistic studies shifting to inflammation, consideration of GPR55 and the inflammasome-3 (Kelley et al., 2019) were addressed experimentally in the dorsal root ganglion (Krames, 2014). Previous studies had indicated that GPR55 played an important role in pain modulation (Schuelert and McDougall, 2011; Staton et al., 2008). Furthermore, earlier data had suggested a proinflammatory role for GPR55 in innate immunity (Chiurchiu et al., 2015). Based on these previous reports and our own observations which demonstrated that paclitaxel elicited increases in GPR55 immunoreactive area in DRG cultures (Brenneman et al., 2022), we decided on GPR55 as the major focus for our neuroinflammation studies relevant to CIPN. In addition, an emerging concept was that chemotherapeutic agents (including paclitaxel) promote inflammatory responses through activation of the NLRP3 inflammasome (Zeng et al., 2019). Our recent studies confirmed the potential role of NLRP3 and IL-1b, critical components of inflammasome-3, in mediating the anti-inflammatory responses of KLS-13019 (Brenneman et al., 2022).

In the studies to be reported, high content fluorescent imaging of dissociated, embryonic day 19-DRG neurons were used to assess target depletion by siRNA in this model system that is relevant to CIPN (Guo et al., 2017). While siRNA knockdown strategies typically use mRNA levels, target immunoreactivity measures from a Western blot analysis or biological attenuation of function as a means of assessing target depletion, the present studies employed a combination of methodologies. With the utilization of high content imaging applied to the diversity and complexity inherent to primary sensory neuron morphology, the goal was to compare the neuronal knockdown of GPR55 immunoreactive spot target area and the attenuation of efficacy for anti-inflammatory actions produced by the two cannabinoids. Importantly, by utilizing the capability of high content imaging, the feasibility of distinguishing and comparing the responses of neurites and cell bodies of sensory neurons in regard to GPR55 immunoreactivity was realized. The overall goal was to assess the relationships between specific cellular locations of target depletion with that of the siRNA-mediated attenuation of anti-inflammatory efficacy produced by the cannabinoid compounds of interest.

In addition to the anti-inflammatory properties of KLS-13019 and CBD, the current studies also examined effects of these cannabinoids on paclitaxel-induced neurite retraction, as neuritic length and area are included in the array of neuronal parameters routinely assayed in high content imaging. Previous studies employing a variety of DRG preparations have indicated that paclitaxel produced significant decreases (> 50%) in neurite length in dorsal root ganglion cultures after 24–48 hours of treatment (Scutteri et al., 2006; Chen et al, 2015). Previous demonstrations of intervention of paclitaxel-induced toxicity and inhibition of neurite outgrowth utilized *pre-treatment* paradigms to demonstrate protection by test compounds. In the studies to be described, DRG cultures were *pretreated* with paclitaxel for 8 hours followed by 16-hour treatment with either CBD or KLS-13019, without the removal of paclitaxel. This strategy was aimed at evaluating possible cannabinoid-induced *reversal* of neurite retraction in DRG neurons produced by paclitaxel. In addition, because these cultures were pre-treated with GPR55 siRNA, it was an accompanying goal to determine if knockdown of this target produced an attenuation of cannabinoid-related reversal from paclitaxel-induced neurite retraction. Indeed, demonstration of the reversibility of both inflammation or neurite retraction from paclitaxel was confirmed with KLS-13019 treatment, but not with cannabidiol.

## Materials and Methods

### Materials

Alamar blue was obtained from Invitrogen (Eugene, OR). Paclitaxel was obtained from Teva Pharmaceuticals USA (Sellersville, PA) as a 6 mg/ml solution containing 527 mg polyoxyl 35 castor oil, 2mg citric acid and 49.7% dehydrated alcohol per ml. Rat NGF-b (N2513) and cannabidiol were obtained from Millipore-Sigma.

The synthesis of KLS-13019 has been described previously in detail (Kinney et al., 2016). Verification of the structural identity for KLS-13019 was determined by ^1^H NMR, ^13^C NMR, HMBC, HSQC, COSY, NOESY, LC/UV, and LC/MS. The purity of KLS-13019 was 98.6% as determined by LC/MS.

### Culture models

Dissociated dorsal root ganglia (DRG) cultures derived from embryonic day 18 rats were employed as the primary assay system to explore anti-inflammatory mechanism of action for KLS-13019 in the context of GPR55 siRNA knockdown studies. In brief, rat DRG were obtained commercially through TransnetYX (Springfield, IL) and cultures prepared according to methods described previously (Brenneman et al., 2019). Tissue was dissociated with a papain-based kit from Worthington Biochemical Corporation (Lakewood, NJ). The DRG cells were plated at low density (10,000 cells / well) in a 96-well format and maintained in serum-free medium consisting of Neurobasal Medium Plus and rat Nerve Growth Factor- b 25 (ng/ml). Poly-D-lysine coated plates (BD Biosciences, Franklin Lakes, NJ) were employed for this culture system. Prior to the initiation of siRNA experiments between days 5 and 9 in vitro, a complete change of medium was performed in a working volume of 100 μL.

GPR55 siRNA treatment was also studied in hippocampal cultures that were prepared by methods previously described (Brenneman et al., 2018). This GPR55-containing culture system was used for preliminary studies to determine optimal concentration ranges of siRNA as well as to assess the time course of siRNA-mediated effects on primary neurons by high content imaging to be described. Hippocampal tissue was obtained commercially through TransnetYX (Springfield, IL). Tissue was dissociated with a papain-based kit from Worthington Biochemical Corporation (Lakewood, NJ). The hippocampal neurons were platted at low density (10,000 cell/well) in a 96-well format and maintained in serum-free medium consisting of Neurobasal Medium Plus (Thermofisher) with B27 Plus Supplement. Poly-L-lysine coated plates (BD Biosciences, Franklin Lakes, NJ) were used for this preparation. Prior to the initiation of siRNA experiments between days 11 and 21 in vitro, a complete change of medium was performed in a working volume of 100 μL.

### GPR55 Knockdown

The ON-targetplus SMARTpool siRNA was obtained from horizondiscovery.com. The compositions of the four pools are described in Table 1. For DRG cultures, the pool of oligonucleotides was added to the cultures on day 5 after plating following a complete change of medium to 100 μl of Accell siRNA delivery medium (Horizon Discovery). For hippocampal cultures, the siRNA was added on day 11 after plating. Preliminary experiments were conducted in the hippocampal cultures because of the more abundant neurons in this preparation than that obtained for DRG cultures. The siRNA treatment paradigm used was as previously described for mNCX-1 knockdown (Brenneman et al, 2019). After a 24-hour treatment with siRNA in Accell medium, the cultures were given a complete change of medium back to Neurobasal Medium Plus (Thermofisher) with the B27 Plus Supplement. The knockdown period duration was 3 days following the removal of Accell medium. For DRG cultures, assessment of the effect of siRNA on GPR55 immunoreactive area was then conduced on fixed cultures by high content imaging on Day 9. The primary purpose of the studies to be described was the assessment of DRG cultures for their inflammatory responses to paclitaxel then to test if the anti-inflammatory actions of KLS-13019 or CBD were attenuated by the GPR55 siRNA knockdown.

### Immunofluorescent assays

To assess the effects of various KLS-13019 treatments, immunofluorescent methods were used to measure neuronal responses in DRG cultures. The goals for these assays included: 1) identification of neuronal structures with antibodies to beta-3 tubulin; 2) to assess the immunoreactive spot area of selected molecular targets (IL-1b, GPR55 and NLRP3) with their respective primary antibodies and distinctively labeled secondary antibodies; 3) to compare the relative responses of the molecular targets in both neuronal cell bodies and neurites; and 4) to assess the effect of GPR55 siRNA on all measures identified in the inflammation assays above.

Prior to fixation, growth medium was removed and the wells were rinsed twice with 100 μL DPBS (37° C). This warm rinse is particularly important to maintain structural stability of neurites. After removal of the DPBS, cultures were fixed for 20 min at room temperature with 50 μL / well of 3.5% formaldehyde (Sigma-Aldrich: 252549) in warm (37°C) DPBS that contained 5.5 μg/mL of Hoechst 33342 dye (Invitrogen: H3570) to label cell nuclei. After removal of the fixative, the cultures were rinsed 3 times with 100 μL of DPBS and then a permeabilization - blocking buffer containing 5% normal goat serum and 0.3% triton-X100 in DPBS was added to the cultures for 10 min. After removal of the blocking buffer, the cultures were rinsed twice with 100 μL of DPBS and then primary antibodies were added for one-hour incubation with shaking at room temperature. Neurons were identified with antiserum to beta-3 tubulin (tuj 1) to measure changes in all neuronal structure parameters. The primary antiserum employed was a rabbit polyclonal obtained from Sigma –Aldrich (T2200) and used at 1:200 dilution. The secondary antibody was an Alexa Fluor 488-conjugated Fab fragment of goat anti-rabbit IgG obtained from Life Technologies (A11070) used at 1:600 for 30 minutes. After the secondary antibody treatment, cultures were rinsed 3 times with 100 μL of DPBS before performing high content fluorescent analysis. For storage prior to image analysis, the wells were placed in 100 μL of sterile DPBS, with the plates wrapped in aluminum foil and maintained at 4° C. For the detection of inflammatory markers, the following primary antibodies were used: IL-1b (PA5–88078); NLRP3 (PA5–79740); and for GPR55 (ab203663). All primary antibodies for IL-1b and NLRP3 were obtained from Life Technologies. The GPR55 antibody was obtained from Abcam. All primary antibodies were diluted 1:250 and all secondary antibodies were used at 1:600. The secondary antibodies were obtained from Life Technologies. The following Alexa Fluor dyes labeled the secondary antibodies: Alexa Fluor 488 (A11070), 555 (A32732), 687(A32733) and 750 nm (A21039). By using secondary antibodies with differing dyes, the same culture wells were assayed for multiple molecular targets.

### High content image analysis

The immunofluorescent assays were conducted on the Cell Insight CX5 high content imaging system (Thermofisher Scientific). The system is based on an inverted microscope that automatically focuses and scans fields of individual culture wells using a motorized stage at predetermined field locations. Fluorescent images from individual fields (895μm × 895μm) were obtained with a 10 × (0.30NA) Olympus objective and Photometrics X1 CCD camera, with analysis by HCS Studio 2.0 Software. The light source was LED with solid state five-color light engine used with filter sets that had the following excitation/emission: 386/440, 485/521, 560/607, 650/694 and 740/809). With this capability, multiple fluorescent assays in a single well were conducted. Images were acquired in a low-resolution mode (4 × 4 binning). Image analyses for neuronal cell bodies and neurites were performed with the Cellomics Neuronal Profiling BioApplication. For analysis of neurons, objects were identified as cells if they had valid nuclei and cell body measures based on size, shape and average intensity. Acceptable ranges were determined in preliminary studies to ensure that aggregated cells and non-cellular objects were excluded from the analysis.

For both the DRG and hippocampal cultures, the goals were to examine the immunoreactive (IR) spot areas for all the analytes of neurons only. Because the neuronal morphology was different between the two cultures, unique size and shape parameters for cell bodies and neuritic arbors were empirically determined for each culture type in preliminary studies. Once these parameters were determined, the analyses for each culture type were used throughout their respective experiments. Important to these analyses, an essential goal was to compare the immunoreactive spot areas for all analytes in both cell bodies and neurites. Beta-3 tubulin immunoreactivity was used to identify the neurons for each culture type (Brenneman et al., 2022). For DRG cultures, ten predetermined fields of view were sampled in each of six replicate wells per plate, with two replicate plates used for each assay. The age of the DRG cultures at the time of analysis was 9 days after plating. Because primary cultures exhibit a variety of neuronal phenotypes and a range of morphological complexities, extensive sampling was employed to obtain an average neuronal response with the inflammatory markers. For measuring parameters of beta-3 tubulin immunoreactivity and spot analysis for the inflammatory markers, the Cellomics Neuronal Profiling Bioapplication was used that combined spot area analysis on neurons that resided within this BioApplication. For analysis of spot immunoreactivity with this BioApplication, a key parameter was the empirical establishment of fluorescent thresholding that permitted the use of a dynamic range that optimized the measurement of fluorescent differences among the treatment groups as well as distinguishing the fluorescent signal from background. This thresholding level was set based on our previous experience with antibody-based assays and the smallest distinguishable size of immunoreactive spots (radius:1.5 μ) under our imaging conditions. With this algorithm, the immunoreactive area was a relative measure that was characterized by an effective computerized spot analysis in a rapid screening mode. Importantly, the same imaging parameters for neurons from all treatment groups were employed for the DRG studies. The key comparisons in these studies were aimed at measuring the changes in immunoreactive area that were associated with cannabinoid compound treatment. Due to the observed differences in the cellular distribution among the analytes, the cellular locations of all inflammatory markers in DRG were determined, thus distinguishing the relative changes between cell bodies and neurites. This capability and experimental focus were obligatory aspects of measuring the inflammatory markers by image analysis. Again, the goal of the studies was to obtain measures of the relative changes in immunoreactive area for each of the analytes after KLS-13019 or CBD treatment with a GPR55 siRNA pretreatment. An estimate of 1000–1300 neurons were assessed for each treatment. Because of our goal to compare and present various assays after treatments, the data were expressed as the percent of control. A table of the control immunoreactive area for each assay is shown in Table 3. In all cases, the results were expressed as immunoreactive area (μ^2^) for each of the analytes per neuron.

### Anti-inflammatory properties of KLS-13019 and GPR55 siRNA

To study the potential effect of KLS-13019 in reversing paclitaxel-induced inflammation, DRG cultures were pretreated with 3 μM paclitaxel to establish inflammatory responses with increases in IL-1b and NLRP3 immunoreactive (IR) spot area (inflammasome-3 marker) being demonstrated. After the establishment of an inflammatory response, KLS-13019 (100 nM) was added to the cultures for an additional 16 hours, with the paclitaxel remaining on the cultures. At the conclusion of the 16-hour treatment period, cultures were fixed and assays conducted as described in the following immunofluorescent assay section. This sequence of 8-hour paclitaxel pretreatment followed by a 16-hour KLS-13019 treatment period will be referred to as the “reversal” paradigm for DRG cultures throughout these studies.

### Anti-inflammatory properties of CBD and GPR55 siRNA

Since CBD has played a prominent role in the discovery and mechanistic history of KLS-13019, comparative studies also were conducted in DRG cultures to investigate the anti-inflammatory activity of this cannabinoid after co-treatment with paclitaxel. The same “reversal” treatment paradigm as that employed for KLS-13019 was employed, except with an increased concentration of CBD. Previous studies (Brenneman et al., 2018) had demonstrated that 10 μM CBD was required to produce a maximal effect on neuroprotection. In the current studies, 10 μM CBD was also employed for the anti-inflammatory studies because higher concentrations produced toxicity in these developing neuronal preparations (Brenneman et al., 2018).

### Calculation of functional attenuation produced by GPR55 siRNA

For all assays and test compounds, the same method of calculating functional attenuation produced by GPR55 siRNA was utilized. The intent was to determine the assay signals that were attributable to only the test agent or only the siRNA, without that portion of the measurement that was attributable to background. The first signal to be calculated was the test agent signal (TAS) which was the difference between value from cultures treated with paclitaxel plus the test agent from the value from cultures treated only with paclitaxel. For these values, the absolute value of this difference was used because some assays decreased and others increased in response to paclitaxel. The test agent signal was used instead of control because one of the test agents (CBD) did not produce complete reversable responses from paclitaxel. The second signal to be determined was the siRNA signal which was determined by the difference between cultures treated by test agent, paclitaxel and siRNA from those cultures treated with test agent and paclitaxel. The % attenuation was then determined by the siRNA signal divided by the TAS: siRNA signal/TAS times 100.

### Statistical Analysis

All statistical comparisons were made by ANOVA, with normality of values tested by the Shapiro-Wilk test followed by a multiple comparison of means test with the Holm-Sidak method as performed through Sigma Plot 16. All error bars are the standard error.

## Results

### GPR55 knockdown: neuronal compartment analysis

The goal of the present studies was to determine if GPR55, an orphan receptor with proposed actions for cannabinoids (Ryberg et al., 2007), was involved in the mechanism of action for the anti-inflammatory drug candidate KLS-13019 (Kinney et al., 2019; Brenneman et al., 2022) and CBD. To explore this possibility, GPR55 siRNA was used to knockdown the IR area of the proposed target and then to ascertain if either the anti-inflammatory efficacies of KLS-13019 or CBD were attenuated in DRG cultures (Brenneman et al., 2022). For these studies, a previously reported treatment paradigm was used to reverse an established inflammatory response produced by the chemotherapeutic agent paclitaxel. In Table 2, two neuronal cultures that express GPR55 are compared for their potency and efficacy in producing siRNA-mediated knockdown of GPR55. Because hippocampal tissue provided a more abundant population of GPR55-positive neurons than DRG tissue, initial studies were screened in cultures derived from hippocampal tissue. In Table 2, it was apparent at 10 nM siRNA that the GPR55 immunoreactive (IR) spots were significantly decreased by 58–62% in comparison to control. This knockdown of GPR55 IR spot area was observed from 4 to 10 days of knockdown duration. Based on the responses of the hippocampal cultures, day 5 DRG cultures were utilized with similar conditions to study a culture system more clearly relevant to CIPN. As shown in Table 2, it was apparent that higher concentrations of GPR55 siRNA were required to achieve similar amounts of GPR55 knockdown for DRG cultures by using 1 μM siRNA. For all the GPR55 siRNA experiments to be described, 1 μM was used for target knockdown. In both primary culture systems, the amount of siRNA-mediated knockdown was significantly greater (58–62%) in neurites than that observed in cell bodies (21–25%).

### Dorsal Root Ganglion Cultures and inflammatory markers

**Figure 1:**
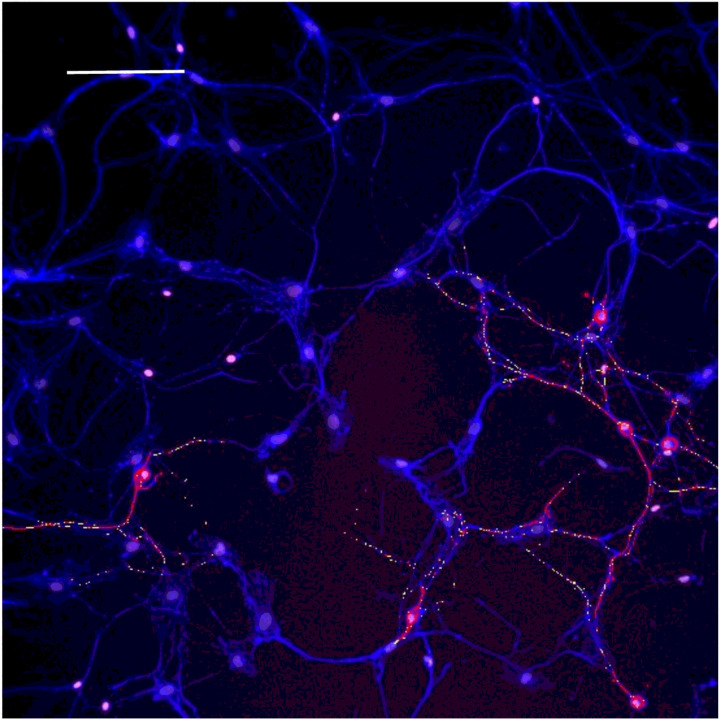
Immunofluorescent image of GPR55 (yellow) and NLRP3 (red) immunoreactivity spots on neurons from Day 9 dorsal root ganglion culture: evidence for co-localization of spots on same neurons DRG neurons (purple) were identified with antibodies to beta-3 tubulin and nuclei are shown as pink spots using Hoechst 33342 dye. The calibration bar = 150 m.

**Figure 2:**
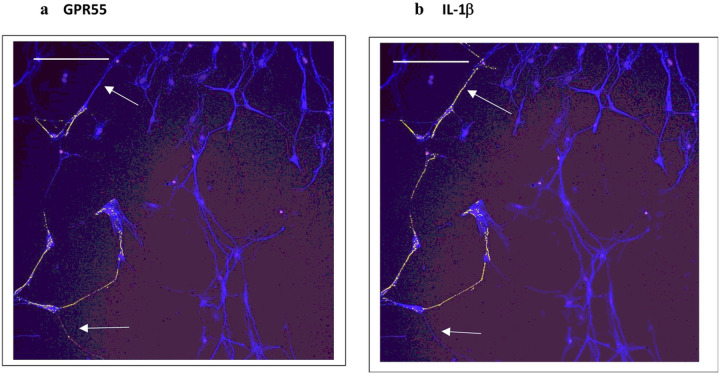
Immunofluorescent images of the same field from dorsal root ganglion cultures comparing GPR55 (a) and IL-1b(b): evidence for slight differences in distribution of GPR55 and IL1b within the same neurons Day 9 DRG neurons were identified with antibodies to beta-3 tubulin. Arrows are pointing to differences in the labeling pattern between GPR55 and IL-1b. The calibration bar = 150 m.

Based on concepts revealed from previous studies of KLS-13019-mediated inhibition of paclitaxel-induced inflammation (Brenneman et al., 2022), the following inflammatory mediators were chosen to monitor effects of GPR55 siRNA: GPR55, NLRP3 and IL-1b. In the present study, the entire focus was on neuronal imaging as determined by beta-3 tubulin immunofluorescence. In Fig. 1, immunofluorescence spots of GPR55 and NLRP3 are shown indicating that these two measures of inflammation tracked together in the same location in GPR55-positive neurons. In all GPR55-positive neurons, NLRP3 immunoreactive (IR) spots were also observed. Most DRG neurons were not IR-positive for either marker, as only 25% of the neurons were positive for GPR55. Because an increase in NLRP3 was suggestive of an activation of the inflammasome − 3 complex, IL-1beta was also surveyed for IR spots in the DRG cultures. As shown in Fig. 2, the IR spot distribution in DRG neurons was compared for GPR55 and IL-1b. As in the observation made in Fig. 1, most neurons were not positive for either GPR55- or IL-1b. However, in all cases, neurons that were positive for GPR55 were also positive for IL-1b, although the distribution within the neurons was often slightly different, with IL-1b being more prevalent. To facilitate comparisons between the various analytes, changes were expressed as a percent of control. In Table 3, the control levels per neuron are presented for each analyte as determined by high content analysis of immunoreactive spots.

### GPR55 knockdown attenuates anti-inflammatory responses to KLS-13019

**Figure 3:**
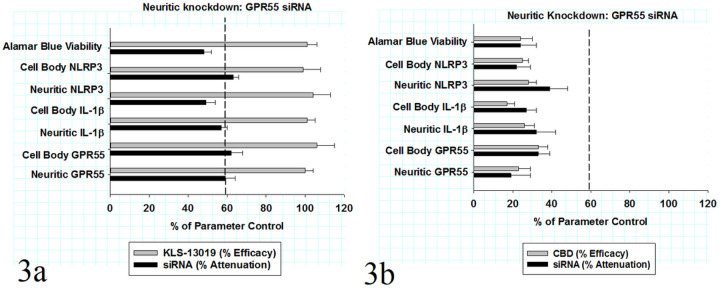
Comparison of KLS-13019 (a) - and CBD (b) -mediated protection from paclitaxel and the attenuation of that protection by GPR55 siRNA pre-treatment in DRG cultures in a 24-hour reversal treatment paradigm Fig.3a: After an 8-hr pretreatment with paclitaxel, DRG cultures were assessed by assays for cell viability (alamar blue) and for six assays of inflammation: GPR55, IL-1b and NLRP3 in both cell bodies and neurites after 16 hours of incubation. All the inflammation assays were conducted by high content fluorescent imaging that utilized antibodies to beta-3 tubulin for neuronal identification. The KLS-13019-mediated reversible protection from paclitaxel-mediated changes are shown by the gray bars as a percentage of their respective parameter control. For all the assays, KLS-13019-mediated protection from paclitaxel resulted in values that were not different from that of control values. The percentage attenuations of the KLS-13019-mediated protection from paclitaxel produced by GPR55 siRNA knockdown are shown by the dark bars. The calculation method for percent attenuation has been described in the methods section. The mean attenuation produced by GPR55 siRNA from all assays was 57 ±2% of their respective responses in cultures treated with 100 nM KLS-13019 and 3 mM paclitaxel. Each value is the mean of twelve well determinations and each of the wells were assessed from 10 fields. Assays were made from duplicate experiments. The vertical dashed reference line depicts the percentage decrease in neuritic GPR55 IR area observed after target knockdown. There were no significant differences in the mean % attenuation among all the assays and the % knockdown of neuritic GPR55 area. Fig 3b: CBD-mediated protection from paclitaxel and the attenuation of that protection by GPR55 siRNA pre-treatment in DRG cultures in a 24-hour reversal treatment paradigm are shown. After an 8-hr pretreatment with paclitaxel, DRG cultures were assessed by the same assays and the same timing as that described in figure 3a. The CBD-mediated reversable protection from paclitaxel-mediated changes is shown by the gray bars as a percentage of their respective parameter control. The mean CBD-mediated protection from paclitaxel for all seven assays was 25 ± 2 % of their respective control values. The percentage attenuations of the CBD-mediated protection from paclitaxel produced by GPR55 siRNA knockdown are shown by the dark bars. The mean attenuation produced by GPR55 siRNA from all assays was 28 ± 3 % of their respective responses in cultures treated with 10 mM CBD and 3 mM paclitaxel. Each value is the mean of twelve well determinations and each well were assessed from 10 fields. Assays were made from duplicate experiments. The vertical dashed reference line depicts the percentage decrease in neuritic GPR55 IR area observed after target knockdown. In the assays of all the CBD-treated cultures, the mean % attenuation produced by GPR55 siRNA was less than one-half of the % knockdown of neuritic GPR55 area.

In all experiments to be described, a reversal paradigm was utilized to detect anti-inflammatory responses in DRG cultures that had been pre-treated for 8 hours with 3 μM paclitaxel to induced inflammatory responses (Brenneman et al., 2022). After the inflammatory responses were established with paclitaxel pre-treatment, KLS-13019 was added to the cultures for additional 16 hours, followed by multi-analyte determinations of the DRG cultures. In addition to the inflammatory markers assayed in fixed cultures, all cultures were assessed before fixation with the viability dye alamar blue to measure possible toxicity and a loss of mitochondrial function (Iuchi et al., 2019). As shown in Fig. 3a, a comparison is made between KLS-13019-mediated reversal from inflammation produced by paclitaxel (grey bars) and by the attenuation of that anti-inflammatory response produced by GPR55 siRNA (black bars). For each inflammatory response, the IR spot areas per neuron for GPR55, NLRP3 and IL-1b were determined in both neuronal cell bodies and the neurites. All of these data were expressed as per cent of control from their respective parameter control values. KLS-13019 was effective in returning all inflammatory assays back to their control levels. The concentration employed for these reversal assays was 100 nM KLS-13019, an amount that was a 10-fold excess to the minimum concentration required for a full anti-inflammatory response. In the black bars, the percent attenuation of the IR spot area for each inflammatory measures are shown for both cell bodies and neurites. The mean attenuation for all six of the inflammatory measures produced by GPR55 siRNA was very similar to one another and not significantly different from the overall mean response of all assays: 56 ± 3%. A dashed vertical reference line is provided to indicate the mean neuritic knockdown (59 ± 6%) of GPR55 IR area from controls. Thus, the attenuation of KLS-13019-mediated responses for anti-inflammatory markers corresponded closely to the knockdown of the neuritic GPR55 immunoreactive target area.

As an additional control, the effect of GPR55 siRNA on paclitaxel-induced induced increases in cell body GPR55 was also measured in the reversal paradigm. These data indicated that a 6 ± 1% attenuation cell body GPR55 IR area was observed in comparison to that from paclitaxel treatment alone (data not shown). It cannot be ruled out that this effect may be an overlapping or additional GPR55 siRNA signal to that observed with co-treatment of KLS-13019 and paclitaxel.

In addition to the inflammatory markers, KLS-13019 was fully effective in returning the Alamar blue viability values back to control levels. Further, the attenuation of KLS-13019-mediated protection from paclitaxel-induced toxicity was 63 ± 7%, a value similar to that observed for the inflammatory responses. Overall, these knockdown data are consistent with the conclusion that loss of IR area produced by siRNA in neurites had a corresponding effect on the efficacy of KLS-13019 in reversing inflammation and cellular toxicity. Together, these data suggest that GPR55 has a significant, if not dominant role in mediating these anti-inflammatory actions produced by KLS-13019.

### GPR55 knockdown and responses to CBD

CBD, the naturally occurring substance upon which the structure of KLS-13019 was based, provided an important and arguably essential comparator on two levels: 1) the parent compound has anti-inflammatory properties based on the literature (Costa et al, 2007; Wang et al., 2023); and 2) CBD did not produce an effective reversal of mechanical allodynia in a mouse model of paclitaxel-induced neuropathic pain (Foss et al., 2021). Thus, utilizing the same reversal paradigm described for KLS-13019, CBD mediated effects were measured with the same assays utilized for the new drug candidate. As shown in Fig. 3b, a comparison is made between CBD-mediated reversal of inflammation produced by paclitaxel (gray bars) and by the attenuation of that anti-inflammatory response produced by GPR55 siRNA (black bars). For each inflammatory response, the IR spot areas per neuron for GPR55, NLRP3 and IL-1b were determined in both neuronal cell bodies and the neurites. All of these data were expressed as per cent of control from their respective parameter control values. CBD was found to be effective in producing a significant effect on all inflammatory assays; however, the mean efficacy for all the assays was only 25 ± 2% of their respective controls. A dashed vertical reference line is provided again to indicate the mean neuritic knockdown of GPR55 IR area from controls: 59 ± 6% of control. In comparison, the mean cell body knockdown of GPR55 IR area was 21 ± 8% of control.

In addition to the inflammatory markers, CBD was only partially effective in returning the alamar blue viability values back to 24 ± 6% of the control level. Further, the *attenuation* of CBD-mediated reversal from paclitaxel-induced decreases was 24 ± 8% of control in the viability assay. Overall, these knockdown data are consistent with the conclusion that the efficacy of CBD in reversing paclitaxel-induced inflammation in the reversal paradigm was 4-fold less than that of KLS-13019. Furthermore, the amount of attenuation of CBD efficacy produced by GPR55 siRNA was less than half of that observed with KLS-13019 under the same experimental conditions.

### Non-targeting siRNA and anti-inflammatory action of KLS-13019

**Figure 4:**
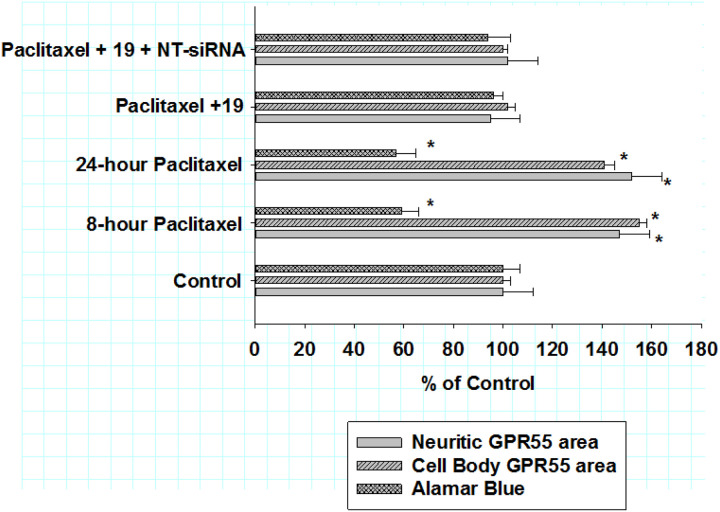
The effects of non-targeting siRNA on the alamar blue viability assay and GPR55 immunoreactive area in DRG cultures co-treated with KLS-13019 and paclitaxel in a reversal paradigm for 24 hours After an 8-hr pretreatment with paclitaxel, DRG cultures were assessed by an assay for cell viability (alamar blue) and for assays of GPR55 both cell bodies and neurites. For the 24-hour treatment group, treatment with 100 nM KLS-13019 was started at 8 hours and continued for another 16 hours, without removal of the pretreatment paclitaxel. The GPR55 assays were conducted by high content fluorescent imaging that utilized antibodies to beta-3 tubulin for neuronal identification. The effect of various treatments on neuritic GPR55 IR area are shown by the solid gray bars. The effects of various treatments on cell body GPR55 IR areas are shown by the single-hatched gray bars. The effect of various treatment on the Alamar blue viability assay are shown by cross-hatched gray bars. All data are presented as % of control of their respective assays. Each value is the mean of twelve well determinations and each of the wells were assessed from 10 fields. Assays were made from duplicate experiments. Significant changes from their respective control values were indicated with an asterisk (P< 0.001). These data indicated that non-targeting siRNA (NT-siRNA) had no detectible effect on the efficacy of KLS-13019 (19) as measured by any of the three assays.

To address the possible incidence for non-specific effects of oligonucleotide treatment on the anti-inflammatory effects of KLS-13019, studies were performed substituting non-targeting siRNA for GPR55 siRNA utilizing the paclitaxel “reversal” paradigm described in the previous section. As shown in Fig. 4, the effect of non-targeting siRNA on three assays were measured: neuritic (solid grey bars) and cell body (gray single-hatched bars) GPR55 spot area as well as the alamar blue viability assay (gray cross-hatched bars). These data confirmed that increases in both cell body and neuritic GPR55 spot area were significantly increased after 8 hr and 24-hr treatments with 3 μM paclitaxel. Importantly, co-treatment with 1 μM non-targeting siRNA produced no attenuation of the anti-inflammatory effects of KLS-13019 on GPR55 IR areas in either cell bodies or neurites. Using the same paradigm, the viability assay utilizing alamar blue was also measured prior to fixation for the GPR55 spot assays. As shown by the cross-hatched bars, paclitaxel treatment produced decreases in the viability assay at both the pre-treatment 8-hour time point and at the 24-hour pre-termination time point. Again, treatment with 100 nM KLS-13019 in this paradigm produced complete reversal of the decreases in the viability assay produced by paclitaxel at 8 hours. Thus, pre-treatment with non-targeting siRNA produced no attenuation of the reversing efficacy of KLS-13019 with this paradigm for any of the assays.

### GPR55 knockdown and neuritic retraction

**Figure 5:**
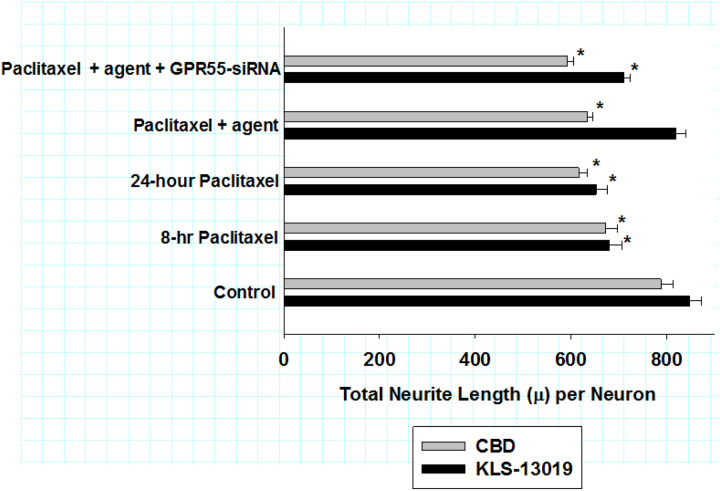
The effects of GPR55 siRNA on total neurite length (m) per neuron are compared between KLS-13019 (black bars) and CBD (gray bars) treatments of day 9 DRG cultures co-treated with paclitaxel in a reversal paradigm for 24 hours. For both sets of experiments, the 8-hr pretreatment values after paclitaxel treatment are shown. DRG cultures were assessed through an assay of total neurite length per neuron as determined by high content imaging. For the agent groups, cultures were co-treated with either 100 nM KLS-13019 or 10 mM CBD. The respective cannabinoid agents were started at 8 hours and continued for another 16 hours, without removal of the pre-treated paclitaxel which was present for 24 hours. Each value is the mean of twelve well determinations, with each well assessed by 10 microscopic fields. Assays were made from duplicate experiments. Significant changes from their respective control values were indicated with an asterisk (P< 0.001). These data indicated that significant neurite retraction (15–19% of control) occurred after 8 hours of 3 mM paclitaxel pre-treatment, with 22–23% neurite retraction being observed after 24 hours of paclitaxel treatment. Cultures co-treated with 100 nM KLS-13019 (19) and paclitaxel had a mean value that was not significantly different from control cultures. In cultures pre-treated GPR55 siRNA, there was a 65 ± 2 % attenuation of the KLS-13019-mediated reversible protection from paclitaxel-mediated neurite retraction. In these cultures, the neuritic knockdown of GPR55 was 59 ± 6 % of control. Thus, the attenuation of KLS-13019-mediated reversal of neuritic retraction was not significantly different from the knockdown of neuritic GPR55 IR area. In contrast, CBD and paclitaxel treatment produced no significant change from cultures treated with paclitaxel alone. Further, GPR55-siRNA treatment of cultures co-treated with paclitaxel and CBD showed no significant effect from cultures treated only with paclitaxel and CBD.

Within the high content analysis algorithm, general neuronal structures characteristics were also analyzed using beta-3 tubulin to identify DRG neuronal structure. These measures indicated that while there was no change in the total number of neurons or cell body areas after the various treatments (data not shown); however, there were significant effects on the total length of neurites per neuron in the day 9 DRG cultures at the termination of the reversal experiment. As presented in Fig. 5, the values for total neurite length per neuron are compared for both KLS-13019 (black bars) and CBD (grey bars) treatment using the reversal paradigm described previously. After the 24-hour treatment period, paclitaxel treatment produced a decrease the total neuritic length per neuron by 21–23% from controls. Treatment with 100 nM KLS-13019 resulted in neurite lengths per neuron that were not significantly different from that of controls. It is of interest that in cultures that had been co-treated with paclitaxel, KLS-13019 and GPR55 siRNA, there was an *attenuation* of the decreased neurite length by 65 ± 2% in comparison to cultures treated with KLS-13019 and paclitaxel. These studies indicate that pharmacological effect of KLS-13019 in reversing a decrease in neurite length could be attenuated by GPR55 knockdown (59 ± 6% of control). In contrast to the reversal effect of KLS-13019, there was no apparent change in total neurite length per neuron after treatment with 10 μM CBD in comparison to cultures treated only with paclitaxel. In addition, cultures treated with paclitaxel, CBD and GPR55 siRNA showed no change in total neurite length per neuron in comparison to that produced by CBD and paclitaxel. Together, these data indicate that KLS-13019, but not CBD, had an effect on reversing paclitaxel-mediated retraction of total neuritic length per neuron.

## Discussion

### Cannabinoids as homeostatic facilitators

A perspective on the therapeutic use of two cannabinoids as facilitators of homeostasis has been addressed in the context of reversing paclitaxel-induced toxicities that occur in cultured DRG neurons. The paclitaxel-induced toxicities that disrupt homeostasis included but were not limited to dysregulation of mitochondrial calcium levels and increases in neuroinflammation from stimulation of the inflammsome-3 mitochondrial system. Our approach has utilized target-specific siRNA to GPR55 for the measurement of the attenuation of the actions for two cannabinoids that have demonstrated differences in efficacy either in preventing or reversing mechanical allodynia in animal models of CIPN (Ward et al, 2011; Foss et al., 2021). With the present studies conducted on DRG cultures and the previous studies conducted by our group with a mouse model of CIPN, a dichotomy of results have been observed in comparing KLS-13019 and CBD. Both compounds can *prevent* mechanical allodynia in the mouse model of CIPN, but only KLS-13019 was observed to *reverse* established mechanical allodynia. The present studies were designed to test if differences in anti-inflammatory properties between KLS-13019 and CBD may relate, in part, to the observed differences in the reversibility of mechanical allodynia in a mouse model of CIPN. Embedded in these studies is the emerging hypothesis that paclitaxel-induced inflammation has a role in mechanical allodynia that is both reversable and treatable with KLS-13019.

The present studies conducted in DRG cultures have shown that KLS-13019 can reverse paclitaxel-induced inflammation, which can be attenuated by GPR55 siRNA. The complete reversal of paclitaxel-mediated increases in NLRP3, GPR55 and IL-1b back to control levels supports that conclusion that KLS-13019 is a homeostatic facilitator. However, the present studies in DRG cultures also indicated that CBD did not have high efficacy in reversing paclitaxel-induced inflammation, a finding which was accompanied by the previous demonstration of a lack of CBD-mediated reversibility in a mouse model of CIPN (Foss et al., 2021).

Previous studies that utilized siRNA to mNCX-1 treatment of DRG cultures focused on the attenuation of the protective properties of both CBD and KLS-13019 in a treatment paradigm that utilized a preventative strategy in which cultures were pre-treated with cannabinoids prior to the addition of paclitaxel. Because mNCX-1 is a key regulator of calcium levels in the mitochondria, this siRNA approach focused on an acute role of cannabinoids in preventing paclitaxel-induced toxicity. These studies demonstrated that both cannabinoids were completely effective in *preventing* paclitaxel-indued toxicity and that theses protective activities were attenuated by and correlated with mNCX-1 knockdown of DRG neurites. Thus, a bimodal molecular target capability has emerged for KLS-13019 in both *preventing* and *reversing* paclitaxel-induced toxicity in DRG neurons; whereas for CBD, there was a more limited pharmacological outcome that was evident only in a preventative therapeutic action.

### Assessment of GPR55 siRNA on anti-inflammatory actions

Our previous investigation of GPR55 began with exploration of paclitaxel-induced changes in GPR55 immunoreactive area in dissociated DRG neurons (Brenneman et al, 2022). These studies indicated that this putative proinflammatory GPCR was increased in IR area within 15–30 minutes of treatment with a clinically relevant amount (3 μM) of paclitaxel. The concept that emerged from this finding is that GPR55 may be among multiple sentinel mechanisms that are induced after exposure to pro-inflammatory drugs or endogenous agonists.

The present study provided evidence for both the comparison of changes in the anti-inflammatory activity associated with two cannabinoids and changes in the immunoreactivity of GPR55 target immunoreactive area in a complex and relevant model of sensory neurons derived from embryonic rat dorsal root ganglion. Of prime importance was to evaluate the attenuation of anti-inflammatory efficacy after GPR55 siRNA treatment. High content fluorescence imaging of the DRG cultures provided necessary technical capabilities for non-biased sampling of the complexities of sensory neuron responses to the GPR55 siRNA treatment. Our studies focused on the immunoreactive area of GPR55 were directed to both cell bodies and neurites as detected by high content fluorescent imaging. As described in Table 2, there was a significant difference in the percentage decrease from controls in GPR55 IR area in cell bodies (21 ± 8%) in comparison to that in neurites (59 ± 6%). This response differential between cell bodies and neurites was similar between both DRG neurons and hippocampal dissociation cultures. In comparison to the knockdown of GPR55 in neurites, the average attenuation produced by GPR55 siRNA for all 6 assays of anti-inflammatory activities from KLS-13019 was virtually the same: 57 ± 3%. These data suggest that the best correlation between target knockdown in the high content analysis and a reduction in anti-inflammatory action KLS-13019 was evident in the neurites. The question remains on why there was an observed decrease of 53 ± 5% in overall anti-inflammatory activity in cell bodies with only an observed 21 ± 6% decrease in GPR55 knockdown area? While the explanation of this difference has yet to be established, we have previously hypothesized that for mNCX-1 the lower percentage decrease in cell bodies was due to the significant heterogeneity of mitochondria in cell bodies. With this explanation, it is hypothesized that some mitochondria in cell bodies have GPR55 that is not accessible to the siRNA treatment because of either developmental immaturity or because of functionally distinct and heterogenous populations of mitochondria in cell bodies. It is noteworthy that a similar disparity between target knockdown of mNCX-1 cell bodies and protective activity from KLS-13019 was reported previously (Brenneman et al., 2019). In the present study, we are evoking the same mitochondrial heterogeneity explanation for the disparity between cell body knockdown of GPR55 and the observed attenuation of cell body anti-inflammatory responses produced by KLS-13019.

### Mitochondrial heterogeneity and cannabinoid responses

As indicated previously, the high content analysis of inflammatory responses to siRNA indicated a regional difference in immunoreactive response, with the neurites being far more responsive to siRNA-mediated knockdown than the cell body mitochondria. Evidence for the heterogeneity of mitochondria between cell bodies and neurites has been reported for DRG neurons (Kuznetsov and Margreiter, 2009). Previous studies (Dedov and Roufogalis, 1999) indicated that the neurite mitochondria were smaller, far more mobile and exhibited a greater mitochondrial membrane potential. Indeed, others have noted that “synaptic mitochondria” located in the neurites were likely involved in the providing support required for neurotransmission and synaptic plasticity (Fedorovich et al., 2017; Kulbe et al., 2017). Emerging from this concept was a hypothesis that these “synaptic mitochondria” may be ultimately the target for KLS-13019-mediated anti-inflammatory and protective activity. With this concept, it would be further hypothesized that the relative population of synaptic mitochondria in cell bodies may be only 1/3 of that in the neurites, roughly equivalent to the relative GPR55 knockdown between the two neuronal compartments. The proposed idea that specialized synaptic mitochondria being the site of drug action may provide a clue about the complex pharmacology for these cannabinoids and the observed response of primary neurons to GPR55 siRNA. Thus, a very close agreement was observed between the loss of GPR55 target area in the neurites and the loss of reversible anti-inflammatory activity associated with KLS-13019 treatment.

Nevertheless, the close correspondence between target knockdown in neuritic mitochondria and the observed decrease in reversable protection does not negate the possibility of other drug targets and non-neuronal cells contributing to the protection provided by these cannabinoids or suggest that mitochondria within the cell bodies do not contribute to the protective mechanism. Rather, the present studies suggest that the GPR55 in synaptic mitochondria are a plausible site for multiple mechanisms of KLS-13019-related actions on sensory neurons after paclitaxel treatment.

### Neurite retraction and GPR55

High content analyses of DRG neurons included general measures of neuronal number, cell body area and neurite-related parameters. Among the later, the studies of paclitaxel-induced changes revealed a small (< 23%) decrease in total neurite length per neuron. Our observed value for paclitaxel-induced neurite retraction in rat DRG cultures was analogous to the previously reported range of 25–75% decreases in neurite outgrowth from controls for mouse DRG cultures treated with paclitaxel (Chen et al., 2015). In the context of our comparisons between two cannabinoid compounds, these studies revealed a consistent differential effect, with KLS-13019 treatment completely preventing the neurite retraction and CBD having no detectible effect on this parameter. The effect of KLS-13019 clearly indicated that the neurite retraction mediated by paclitaxel was a reversable effect, which was attenuated (65 ± 2%) by GPR55 siRNA. The observed effect of GPR55 siRNA was not anticipated and remains to be explained in terms of either an inflammation-related effect or by some other regulatory/toxic process. While the explanation for this effect is not yet clear, it is of interest that in PC12 cells, lysophosphatidylinositol, an endogenous agonist of GPR55, has been shown to produced neurite retraction (Obara et al., 2011). In the event that this neurite retraction response to LPI occurs in DRG neurons, it is likely that the neurite retraction is related also to an inflammatory event through the pro-inflammatory GPR55 target. In addition, GPR55 has been detected on axons and growth cones from retinal ganglion explants (Cherif et al., 2015), suggesting a broader scope of action on pharmacologically-mediated effects on neuronal processes.

#### Summary

These GPR55 siRNA studies support a pro-inflammatory role for GPR55 in DRG cultures and confirm a role for this GPCR in the anti-inflammatory properties of KLS-13019, a known GPR55 antagonist (Brenneman et al., 2022). The GPR55 target is believed to be complementary to our previous studies with the NCX-1 target which demonstrated that the expression of this mitochondrial sodium-calcium exchanger was obligatory for protection from paclitaxel in DRG cultures (Brenneman et al. 2019).

Together, our data indicate a bimodal pharmacological effect of KLS-13019 that can be both increase viability of sensory neurons exposed to paclitaxel and antagonize GPR55-mediated neuroinflammation that may contribute to neuropathic pain. Furthermore, the present studies suggest that the significant differences between the anti-inflammatory properties of KLS-13019 and CBD may relate, in part, to their observed differences in the reversibility of mechanical allodynia in a mouse model of CIPN. The emerging concept to be further investigated is the fundamental role of neuroinflammation in mediating neuropathic pain in CIPN and the therapeutic relevancy of GPR55 antagonism in treating this unmet medical need.

## Figures and Tables

**Table 1:** Composition of On-target GPR55 siRNA pool

siRNA pool components	Sequence
1	CUACCAAUCUUGUCGUCUU
2	CUAUAGGAGCAUUCACAUU
3	CUUCUAUCUACAUGAUCAA
4	CGUUCGUUUUCGUGGACAA

**Table 2:** Summary of GPR55 Knockdown in Hippocampal and Dorsal Root Ganglion Cultures

Duration of Knockdown	Culture Source	% Decrease in GPR55 Area		siRNA Concentration^1^	
		Cell Bodies	Neurites	Cell Bodies	Neurites
4 days	Hippocampus	20 ±5	58 ± 8	10 nM	1 nM
7 days	Hippocampus	19 ± 5	62 ± 6	10 nM	10 nM
10 days	Hippocampus	25 ±3	58 ± 7	1 nM	1 nM
4 days	Dorsal Root Ganglion	21 ±8	59 ± 6	100 nM	1000 nM

1 Minimum concentration required to produce maximum knockdown.

**Table 3:** Day 9 dorsal root ganglion cultures: comparison of immunoreactive areas from various assays of controls^1^

Assay	Control level Areas		Neurite/Cell Body Ratio
Assay	m2 / neuron		
	Cell Bodies	Neurites	
GPR55	149 ± 10	220 ± 13	1.5
NLRP3	94 ± 4	254 ± 21	2.7
IL-1b	68 ± 3	132 ± 10	1.9
b-3 tubulin	488 ± 10	4929 ± 230	10.1

1 Values were taken from two experiments with mean control values analyzed from 1320 neurons
